# Attribution of Adverse Events Following Coronary Stent Placement Identified Using Administrative Claims Data

**DOI:** 10.1161/JAHA.119.013606

**Published:** 2020-02-16

**Authors:** Sanket S. Dhruva, Craig S. Parzynski, Ginger M. Gamble, Jeptha P. Curtis, Nihar R. Desai, Robert W. Yeh, Frederick A. Masoudi, Richard Kuntz, Richard E. Shaw, Danica Marinac‐Dabic, Art Sedrakyan, Sharon‐Lise T. Normand, Harlan M. Krumholz, Joseph S. Ross

**Affiliations:** ^1^ University of California, San Francisco, School of Medicine and San Francisco Veterans Affairs Healthcare System San Francisco CA; ^2^ National Clinician Scholars Program Yale School of Medicine New Haven CT; ^3^ Center for Outcomes Research and Evaluation Yale–New Haven Hospital New Haven CT; ^4^ Section of Cardiovascular Medicine Department of Medicine, and National Clinician Scholars Program Yale School of Medicine New Haven CT; ^5^ Richard A. and Susan F. Smith Center for Outcomes Research in Cardiology Boston MA; ^6^ Division of Cardiovascular Medicine Beth Israel Deaconess Medical Center Boston MA; ^7^ Harvard Medical School Boston MA; ^8^ Baim Institute for Clinical Research Boston MA; ^9^ Division of Cardiology Department of Medicine University of Colorado Anschutz Medical Campus Aurora CO; ^10^ Medtronic, Inc. Minneapolis MN; ^11^ Department of Clinical Informatics California Pacific Medical Center San Francisco CA; ^12^ Office of Clinical Evidence and Analysis Center for Devices and Radiological Health U.S. Food and Drug Administration Silver Spring MD; ^13^ Department of Health Policy and Research Weill Cornell Medicine New York Presbyterian Hospital New York NY; ^14^ Department of Health Care Policy Harvard Medical School Boston MA; ^15^ Department of Biostatistics Harvard T.H. Chan School of Public Health Harvard University Boston MA; ^16^ Department of Health Policy and Management Yale School of Public Health New Haven CT; ^17^ Section of General Medicine Department of Medicine, and National Clinician Scholars Program Yale School of Medicine New Haven CT

**Keywords:** drug‐eluting stent, percutaneous coronary intervention, real‐world data, registry, surveillance, Health Services, Quality and Outcomes, Percutaneous Coronary Intervention, Stent, Revascularization

## Abstract

**Background:**

More than 600 000 coronary stents are implanted during percutaneous coronary interventions (PCIs) annually in the United States. Because no real‐world surveillance system exists to monitor their long‐term safety, claims data are often used for this purpose. The extent to which adverse events identified with claims data can be reasonably attributed to a specific medical device is uncertain.

**Methods and Results:**

We used deterministic matching to link the NCDR (National Cardiovascular Data Registry) CathPCI Registry to Medicare fee‐for‐service claims for patients aged ≥65 years who underwent PCI with drug‐eluting stents (DESs) between July 1, 2009 and December 31, 2013. We identified subsequent PCIs within 1 year of the index procedure in Medicare claims as potential safety events. We linked these subsequent PCIs back to the NCDR CathPCI Registry to ascertain how often the revascularization could be reasonably attributed to the same coronary artery as the index PCI (ie, target vessel revascularization). Of 415 306 DES placements in 368 194 patients, 33 174 repeat PCIs were identified in Medicare claims within 1 year. Of these, 28 632 (86.3%) could be linked back to the NCDR CathPCI Registry; 16 942 (51.1% of repeat PCIs) were target vessel revascularizations. Of these, 8544 (50.4%) were within a previously placed DES: 7652 for in‐stent restenosis and 1341 for stent thrombosis. Of 16 176 patients with a claim for acute myocardial infarction in the follow‐up period, 4446 (27.5%) were attributed to the same coronary artery in which the DES was implanted during the index PCI (ie, target vessel myocardial infarction). Of 24 288 patients whose death was identified in claims data, 278 (1.1%) were attributed to the same coronary artery in which the DES was implanted during the index PCI.

**Conclusions:**

Most repeat PCIs following DES stent implantation identified in longitudinal claims data could be linked to real‐world registry data, but only half could be reasonably attributed to the same coronary artery as the index procedure. Attribution among those with acute myocardial infarction or who died was even less frequent. Safety signals identified using claims data alone will require more in‐depth examination to accurately assess stent safety.

**Clinical Perspective**

**What Is New?**

- Claims data are often used for postmarket surveillance of coronary stents.
- The extent to which adverse events (repeat percutaneous coronary intervention, myocardial infarction, and death) identified with claims data can be attributed to a previously placed stent is uncertain.
- By linking Medicare claims with a national registry of percutaneous coronary interventions, we found that only half of repeat percutaneous coronary interventions, one‐fourth of myocardial infarctions, and 1% of deaths identified with claims data within 1 year after index percutaneous coronary intervention could be attributed to a previously placed coronary drug‐eluting stent.

**What Are the Clinical Implications?**

- Although real‐world data sources, such as claims, are increasingly important for longitudinal postmarket surveillance of medical devices, including coronary stents, claims data alone may be insufficient to ascertain stent safety and may only serve as a signal for further evaluation.
- Postmarket surveillance would be strengthened with complementary data sources, in addition to claims, to evaluate stent safety.

## Introduction

In recent years, the US Food and Drug Administration has increasingly shifted toward a life‐cycle regulatory approach for medical devices,^1^, ^2^ allowing the agency more flexibility in premarket clinical trial requirements with greater reliance on longitudinal postmarket surveillance to confirm and continue to reassess safety and effectiveness. The recent creation of the National Evaluation System for health Technology is intended to promote the use of real‐world evidence to support medical device regulatory evaluations,^3^ and the US Food and Drug Administration recently released a Guidance Document^4^ about how real‐world evidence can support medical device regulatory decision making. An important and frequently used source of data for these purposes is administrative claims,^5^ which are billing data collected by health plans that include basic demographic and clinical information, as well as longitudinal information on clinical encounters, as long as patients have continuous coverage with the same health plan aggregating the claims. Although their ubiquity makes claims data attractive,^6^ they have important limitations, not unlike other real‐world evidence sources.^5^ These data are not principally designed to support research^5^; lack detailed clinical information; and may not include all of the diagnoses or procedures performed during a hospitalization or clinic visit.^7^ With respect to safety surveillance, claims data have been used with the hope that relevant outcomes can be identified from claims and be reasonably attributed to medical device performance. For claims data to be useful for this purpose, it is critical to know whether adverse events identified using claims data can be reasonably attributed to the medical device in question.

Coronary stents play a key role in the revascularization of patients with coronary artery disease. In 2014, over 667 000 percutaneous coronary interventions (PCIs), >90% of which include stent placement,^8^ were recorded in the National Cardiovascular Data Registry (NCDR) CathPCI Registry,^9^ which includes >90% of PCI‐capable hospitals in the United States. However, important safety concerns pertaining to coronary stents have been discovered since their original approval, including late‐stent thrombosis among drug‐eluting stents (DESs),^10^ higher thrombosis and myocardial infarction (MI) risk associated with bioresorbable vascular scaffolds,^11^ and in‐stent restenosis, a progressive narrowing from vascular remodeling and neointimal hyperplasia.^12^ Despite these concerns, coronary stent surveillance has been challenging because there is no established surveillance system and it is unknown how often the clinical sequelae of these safety‐related adverse events that can be identified in claims data, such as need for repeat coronary revascularization, can be reasonably attributed to a previously placed stent.

Coronary stents thus offer a unique opportunity to better understand the utility of claims data to characterize medical‐device–related adverse events because they are commonly implanted and existing data sources containing detailed information on coronary stent implantations have been linked to administrative claims. Specifically, the NCDR CathPCI Registry includes detailed patient, clinical, and procedural information—including coronary artery–level data—for patients receiving PCI. These data have been linked to longitudinal claims data from the Centers for Medicare and Medicaid Services (CMS) to allow identification of adverse events, which can, in turn, be evaluated to determine whether they are related to previous coronary stent placement.

Accordingly, we sought to assess the extent to which a repeat PCI identified using claims data could be reasonably attributed to the same coronary artery in which a coronary stent was first implanted (ie, target vessel revascularization [TVR]). We focused on TVR because it is an end point frequently used to assess coronary stent safety postimplantation. We did this by identifying index DES placements from the NCDR CathPCI Registry, characterizing incidence of safety‐related adverse events during 1 year of patient follow‐up using Medicare fee‐for‐service (FFS) claims data and then linking those patients who experienced safety‐related adverse events in claims back to the NCDR CathPCI Registry. We also sought to understand what characteristics are associated with greater attribution of a repeat PCI to the artery that had previously received a stent, given that those factors could help target surveillance efforts. We focused on DES given that the vast majority of PCIs involve DES placement. Results from this study can inform our ability to use claims data for ascertainment of stent safety as a part of real‐world postmarket device surveillance.

## Methods

This study was approved by the Yale University Human Investigation Committee; informed consent for the purpose of this project was not required. The study was approved by the NCDR and the CathPCI Research & Publications Committee reviewed the final manuscript before submission, but had no role in the design, conduct, or reporting of the study. Requests to access the CathPCI data that were used for this study can be sent to the American College of Cardiology's NCDR at ncdr@acc.org; https://cvquality.acc.org/NCDR-Home/registries/hospital-registries/cathpci-registry.

### Data Sources

The CathPCI Registry, an initiative sponsored by the American College of Cardiology Foundation and the Society for Cardiovascular Angiography and Interventions, is the largest PCI registry in the United States, used in >90% of PCI‐capable hospitals^9^ and has been described previously.^13^, ^14^ Data on patient demographics, comorbidities, episode of care, and procedural data are included from hospitalization during which PCI is performed. A data quality program ensures reliable and consistent data.^15^ All data elements are recorded by trained abstractors and electronically forwarded to a secure data server. Institutions had to meet NCDR quality criteria for reporting to be included.

We identified longitudinal outcomes in inpatient and outpatient institutional claims for Medicare FFS beneficiaries using *International Classification of Diseases*,* Ninth Revision*,* Clinical Modification* (*ICD‐9‐CM*) codes. These data sets contain claims for inpatient admissions and outpatient care, including procedures for Medicare FFS patients. Additionally, we used 2009–2014 Medicare Beneficiary Summary Files to obtain FFS enrollment and the postdischarge vital status of each beneficiary. We linked Medicare inpatient and outpatient institutional claims to the NCDR CathPCI Registry using deterministic matching on Social Security number, date of birth, and sex.

### Study Population

**Figure 1:**
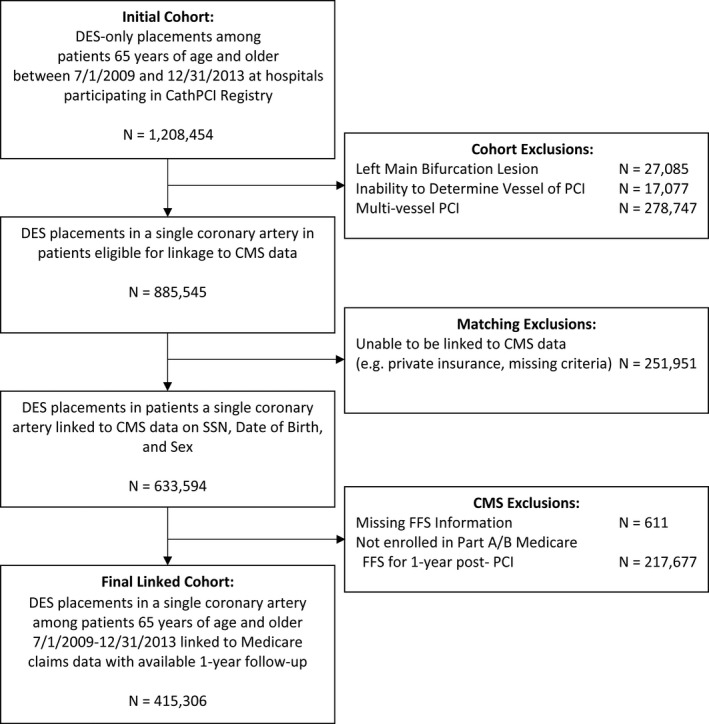
Flow diagram of included drug‐eluting stent placements. BMS indicates bare‐metal stent; CMS, Centers for Medicare and Medicaid Services; DES, drug‐eluting stent; FFS, fee‐for‐service; PCI, percutaneous coronary intervention; SSN, Social Security number.

We identified all patients aged ≥65 years who underwent a DES implantation from July 1, 2009 and December 31, 2013, were linked to CMS claims data, and who were continuously enrolled in FFS Part A and B for 1 year following the index procedure or until the date of death if they died within the year after the procedure (Figure 1). Patients aged ≥65 years who cannot be linked to CMS claims data are likely enrolled in the Medicare Advantage program.^16^ We excluded patients who received both a bare‐metal stent and a DES, multiple DESs at 1 visit in different coronary arteries, or a single DES that crossed >1 of the 4 major epicardial coronary arteries (left main, left anterior descending, left circumflex, and right coronary artery) because attribution to a single stent in a single vessel would be more challenging. We included patients who received multiple DESs at 1 visit within the same coronary artery as a single implant.

### Outcomes and Definitions

We aimed to characterize the proportion of repeat PCIs within 1 year of index DES implantation that represented TVR. We chose to focus first on TVR, instead of target lesion revascularization, because lesion information may be inconsistently reported, particularly for patients who receive multiple procedures from multiple providers.

We first identified subsequent PCIs in CMS claims in the year after an index PCI using *ICD‐9‐CM* procedure codes or Current Procedural Terminology codes (see Table S1 for *ICD‐9‐CM*/CPT codes). We subsequently linked these claims back to the CathPCI Registry to determine which vessel was revascularized. For all analyses, we defined TVR as an unstaged repeat PCI performed in the same vessel treated during the index PCI. Branch vessels were all collapsed to the primary epicardial coronary artery because our analysis was conducted at the vessel level (eg, diagonal vessels were considered part of the left anterior descending system and obtuse marginals were considered part of the left circumflex system). We considered a repeat PCI staged if it occurred within 60 days of the index PCI given that ≤25% of staged PCIs occur >1 month after index PCI,^17^ and did not have a primary discharge diagnosis code of myocardial infarction (MI) or any other diagnostic code suggesting a procedural complication. We still followed patients identified as having a staged PCI after 60 days for subsequent revascularization, MI, or death within 1 year of the index procedure.

Among those identified as TVR, we also examined whether the repeat PCI was performed on a specific lesion that had been previously stented with a DES and, if so, we categorized it as attributable to either in‐stent restenosis or stent thrombosis.

We also characterized MI and death in the year following the index PCI.^18^ Although many causes of MI or death are not likely stent related, we still examined both because these are the most important clinical events that can be secondary to stent‐related complications, and these are the types of adverse events commonly used by regulators to ascertain medical product safety. We identified MI using *ICD‐9‐CM* primary discharge diagnosis codes during a subsequent hospitalization. We obtained dates of death from the master summary beneficiary file.

For patients for whom repeat revascularization and MI events were identified from Medicare claims, we further sought to understand whether baseline patient demographic, clinical, and procedural characteristics were associated with successful attribution to the same coronary artery in which a DES had been previously placed.

### Patient, Clinical, and Procedural Characteristics of Interest

We identified multiple patient, clinical, or procedural characteristics of interest (Table 1). These included patient demographics, cardiovascular history, and other relevant clinical history. We also included procedural and hospital characteristics from the episode of care associated with the original PCI and stent implantation, as well as characteristics of the coronary artery in which the stent was implanted. If 2 DESs were placed in the same artery, their lengths were added, ignoring potential overlap between stents. Among previously treated lesions, we determined whether previous PCI included a stent and, if so, whether the index procedure was performed for in‐stent restenosis or in‐stent thrombosis.

**Table 1:** Patient, Procedural, and Vessel Characteristics

	No.	%
N procedures	415 306	
Demographics		
Age, y, mean (SD)	74.23	6.56
Sex: female	158 086	38.06
Race		
White	381 545	91.87
Black	21 220	5.11
Asian	6022	1.45
Other	6519	1.57
Hispanic or Latino ethnicity	14 571	3.51
Cardiovascular history		
Previous MI	127 114	30.62
Previous HF	60 661	14.61
Previous valve surgery/procedure	7984	1.92
Cerebrovascular disease	67 995	16.38
Peripheral arterial disease	66 436	16.00
NYHA class (among those with HF in previous 2 wk)		
I	4674	10.22
II	13 751	30.06
III	17 451	38.15
IV	9866	21.57
Cardiomyopathy or left ventricular systolic dysfunction	47 157	11.36
Cardiogenic shock w/in 24 h	4642	1.12
Cardiac arrest w/in 24 h	4303	1.04
Other clinical history		
Current/recent smoker (w/in 1 y)	53 226	12.82
Hypertension	363 037	87.44
Dyslipidemia	347 251	83.68
Currently on dialysis	9598	2.31
Chronic lung disease	73 070	17.60
Diabetes mellitus	157 748	38.00
Procedure characteristics		
CAD presentation		
No symptoms, no angina	37 350	9.00
Symptoms unlikely to be ischemic	12 652	3.05
Stable angina	79 185	19.07
Unstable angina	176 881	42.60
NSTEMI	70 514	16.98
STEMI or equivalent	38 639	9.31
Previous PCI	190 461	45.87
Previous CABG	98 158	23.64
Diagnostic Cath status		
Elective	177 098	50.68
Urgent	129 045	36.93
Emergency	42 815	12.25
Salvage	472	0.14
Procedure year		
2009	43 280	10.42
2010	100 041	24.09
2011	89 269	21.49
2012	91 086	21.93
2013	91 630	22.06
PCI status		
Elective	208 464	50.22
Urgent	162 300	39.10
Emergency	43 759	10.54
Salvage	611	0.15
IABP	5240	1.26
Other mechanical ventricular support	1381	0.33
Multivessel disease	208 373	50.17
Vessel characteristics		
No. of stents placed, median (IQR)	1.00	(1.00–2.00)
Sum of stent diameter, median (IQR)	3.00	(2.75–5.00)
Sum of stent length, median (IQR)	22.00	(15.00–33.00)
Stent length categorized		
Short (≤16 mm)	141 135	34.02
Medium (>16–28 mm)	146 361	35.28
Long (>28 mm)	127 332	30.70
Mean vessel stenosis before Tx, median (IQR)	90.00	(80.00–95.00)
Preprocedure TIMI flow		
TIMI—0	37 967	9.17
TIMI—1	32 786	7.92
TIMI—2	82 852	20.00
TIMI—3	260 598	62.92
Previously treated lesion	50 666	12.21
Among previously treated lesions		
Previously treated lesion time frame		
<1 mo	1994	3.94
1 to 5 mo	5668	11.21
6 to 12 mo	6370	12.60
1 to 2 y	7209	14.26
>2 y	25 365	50.16
Time unknown	3959	7.83
Treated with stent	47 152	93.19
In‐stent restenosis	43 445	92.19
In‐stent thrombosis	4613	9.80
Lesion in graft		
Not in graft	377 850	91.02
Vein	34 644	8.35
LIMA graft	1854	0.45
Other artery	795	0.19
Lesion complexity		
Non‐high/non‐C	190 068	45.80
High/C	224 936	54.20
Maximum lesion length, median (IQR), mm	18.00	(12.00–24.00)
Thrombus present	41 432	9.98
Bifurcation lesion	50 592	12.19

CABG indicates coronary artery bypass graft; CAD, coronary artery disease; Cath, catheterization; HF, heart failure; IABP, intra‐aortic balloon pump; IQR, interquartile range; LIMA, left internal mammary artery; MI, myocardial infarction; NSTEMI, non–ST‐segment–elevation myocardial infarction; NYHA, New York Heart Association; PCI, percutaneous coronary intervention; STEMI, ST‐segment–elevation myocardial infarction; TIMI, thrombolysis in myocardial infarction; Tx, treatment.

### Statistical Analysis

We estimated the proportion of patients for whom potential stent‐related safety events (TVR, MI, and death) identified from Medicare claims could be attributed to the same coronary artery in which a DES had been previously placed, overall and stratified by the individual safety end points: TVR, MI, and death. For patients with multiple safety events identified after stent implantation, we analyzed events separately. We performed a sensitivity analysis for patients without a history of previous PCI. We used χ^2^ tests for categorical variables and Wilcoxon or *t* tests for continuous variables. We considered comparisons significant at *P*<0.05. For analyses examining whether baseline patient demographic, clinical, and procedural characteristics were associated with successful attribution, we did not correct for multiple comparisons because these analyses were considered exploratory.

## Results

### Study Cohort

Between July 1, 2009 and December 31, 2013, 919 636 patients aged ≥65 years identified in the CathPCI Registry received a total 1 208 454 DES placements during 1 056 056 PCI procedures (Figure 1). We excluded 27 085 DES placements for left main bifurcation lesions, 17 077 multivessel PCIs with inability to identify which vessel received the DES, and 278 747 in which multiple DESs were implanted into >1 coronary artery. Next, we excluded 251 951 DES placements in patients who could not be linked to CMS data for longitudinal follow‐up (this includes patients enrolled in Medicare Advantage plans, Medicaid plans, other state‐sponsored plans, or employer‐based insurance), 611 without CMS FFS information, and 217 677 who did not have continuous Part A and B FFS enrollment for 1 year post‐PCI. Our final sample of NCDR‐Medicare FFS‐linked data included 415 306 index DES placements in 368 194 patients at 1380 hospitals.

### Patient and Procedural Characteristics

Mean age of patients undergoing PCI in our cohort was 74.2 years, 38.1% were female, and 91.9% were white (Table 1). Furthermore, 30.6% of patients had a history of previous MI, 45.9% previous PCI, and 23.6% previous coronary artery bypass graft. Unstable angina was coronary artery disease presentation in 42.6%, non–ST‐segment–elevation myocardial infarction in 17.0%, and ST‐segment–elevation myocardial infarction (STEMI) in 9.3%. At the index PCI, a median of 1 stent was placed (interquartile range, 1–2), with median stent length of 22 mm (interquartile range, 15–33). Twelve percent of lesions had been previously treated, half of which occurred >2 years before the index PCI; of these previously treated lesions, 92.2% were for in‐stent restenosis and 9.8% for in‐stent thrombosis.

Of the 415 306 DES placements in 368 194 patients, 61 409 (14.8%) were found to have had any adverse event identified within CMS claims data within 1 year: repeat PCI, MI, or death. Of these, 28 607 (46.6%) were successfully linked back to the NCDR CathPCI Registry, including angiographic data, because the patients underwent coronary angiography and/or PCI at the time of the adverse event. Patients for whom angiographic data could be linked were younger and more often male, less commonly had heart failure, cerebrovascular disease, cardiogenic shock, cardiac arrest, presentation with STEMI, and were less often urgent or emergency procedures compared with patients for whom angiographic data were unavailable (Table S2).

### Adverse Events Linked From CMS Claims to the NCDR CathPCI Registry

#### Target vessel revascularization

**Figure 2:**
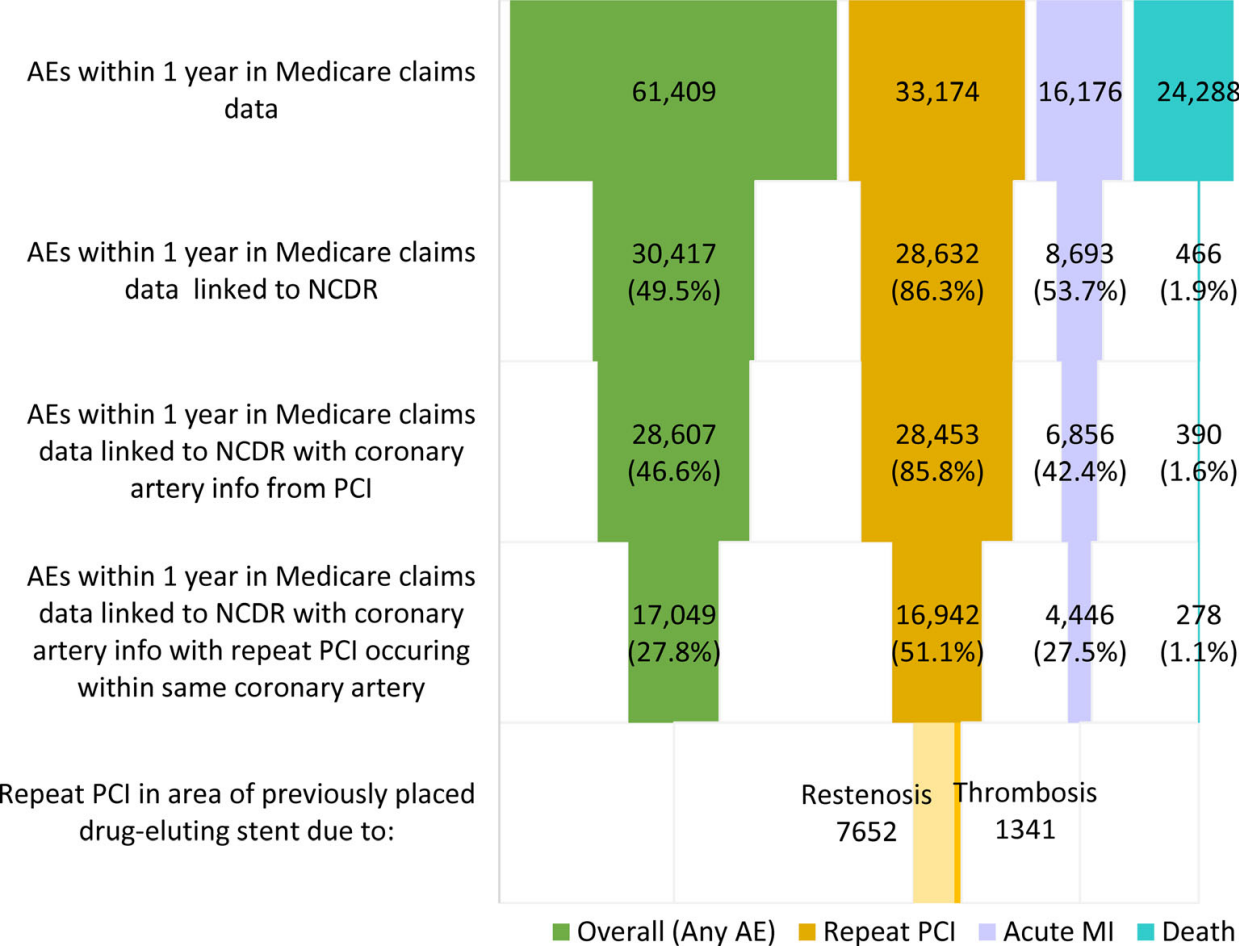
Attribution of adverse events identified in Medicare claims data after index drug‐eluting stent placement in patients aged ≥65 years, 2009–2013. AEs indicates adverse events; MI, myocardial infarction; NCDR, National Cardiovascular Data Registry; PCI, percutaneous coronary intervention.

Of the 415 306 DES placements in 368 194 patients, 33 174 (8.0%) were followed by repeat PCIs identified in CMS claims data (not considered staged PCIs) within 1 year (Figure 2). Of these, 28 632 (86.3%) were successfully linked back to NCDR CathPCI Registry data because the patient received diagnostic coronary angiography and/or PCI, 28 453 (85.8%) included data on coronary anatomy, and 16 942 (51.1%) were attributed to the same coronary artery treated during the index PCI (ie, TVR). Of the 16 942 TVRs, 9954 (58.8%) could be identified as having been inserted within a previously placed stent; of these, 1410 were previously placed bare metal stents and 8544 (50.4%) were previously placed DESs. Among the latter, 7652 were TVRs for in‐stent restenosis and 1341 for stent thrombosis. Overall results of the proportion of patients with TVR were consistent in a sensitivity analysis of patients without a history of previous PCI (Figure S1).

#### Myocardial infarction

Of the 415 306 DES placements in 368 194 patients, 16 176 (3.9%) were followed by acute MIs identified in CMS claims data within 1 year (Figure 2). Of these, 8693 (53.7%) were successfully linked to NCDR CathPCI Registry data because the patient received diagnostic coronary angiography and/or PCI, 6856 (42.4%) included data on coronary anatomy from PCI, and 4446 (27.5%) were attributed to the same coronary artery in which the stent was implanted during the index PCI (ie, target vessel MI), and 2410 could not be attributed to that same coronary artery. In total, of the 6856 DES placements that were followed by acute MIs identified in CMS claims data with NCDR CathPCI data available on patients’ coronary anatomy from PCI, 4446 (64.8%) were attributed to the same coronary artery in which the stent was implanted during the index PCI.

#### Death

Of the 415 306 DES placements in 368 194 patients, 24 288 (5.8%) were followed by patient death identified in CMS claims data within 1 year. Of these, 466 (1.9%) could be successfully linked to NCDR CathPCI Registry data because the patient received diagnostic coronary angiography and/or PCI, 390 (1.6%) included data on coronary anatomy from PCI, and 278 (1.1%) were attributed to the same coronary artery in which the DES was implanted during the index PCI, and 112 could not be attributed to that same coronary artery. In total, of the 390 DES placements that were followed by patient death identified in CMS claims data with NCDR CathPCI data available on coronary anatomy from PCI, 278 (71.3%) were attributed to the same coronary artery in which the stent was implanted during the index PCI.

### Association Between Index PCI Characteristics and Attribution of Repeat PCI or MI

In exploratory analyses, several patient and procedural characteristics were associated with higher rates of successful attribution of a repeat PCI or MI identified from claims data to the same coronary artery as the originally placed DES, including previous MI, previous PCI before index stent placement, and previous coronary artery bypass grafting (Tables 2 and 3). Time from index to repeat PCI was also significantly associated with attribution: PCIs and MIs identified before 30 or >90 days after index PCI were more frequently attributed compared with PCIs between 31 and 90 days (repeat PCI: 54.6% for <30 days versus 37.5% between 31 and 90 days versus 53.4% >90 days after index PCI; *P*<0.001). Total length of stent(s) placed at index PCI >28 mm was associated with greater attribution compared with shorter total stent lengths (repeat PCI: 53.5% for >28 mm versus 50.3% for ≤16 mm and 49.3% for >16–28 mm; *P*<0.001).

**Table 2:** Association of Patient, Procedural, Vessel, and Hospital Characteristics With Attribution of Repeat PCI Events to Index Vessel

	Overall		Not Attributed		Attributed		*P* Value
	No.	%	No.	%	No.	%	
N procedures	33 174		16 232	48.93	16 942	51.07	
Demographics							
Age, y, mean (SD)	73.86	6.36	73.95		73.77		0.020
Sex: female	12 234	36.88	6035	49.33	6199	50.67	0.265
Race							0.007
White	30 225	91.11	14 752	48.81	15 473	51.19	
Black	1915	5.77	924	48.25	991	51.75	
Asian	495	1.49	277	55.96	218	44.04	
Other	539	1.62	279	51.76	260	48.24	
Cardiovascular history							
Previous MI	12 701	38.30	5854	46.09	6847	53.91	<0.001
Previous HF	5803	17.50	2733	47.10	3070	52.90	0.002
Cerebrovascular disease	6662	20.09	3115	46.76	3547	53.24	<0.001
Peripheral arterial disease	6909	20.83	3101	44.88	3808	55.12	<0.001
NYHA class (among those with HF in past 2 wk)							0.545
I	396	10.66	197	49.75	199	50.25	
II	1132	30.48	567	50.09	565	49.91	
III	1415	38.10	670	47.35	745	52.65	
IV	771	20.76	372	48.25	399	51.75	
Cardiomyopathy or left ventricular systolic dysfunction	3969	11.97	1893	47.69	2076	52.31	0.097
Other clinical history							
Current/recent smoker (w/in 1 y)	3781	11.40	1972	52.16	1809	47.84	<0.001
Hypertension	30 198	91.06	14 671	48.58	15 527	51.42	<0.001
Dyslipidemia	28 999	87.49	13 969	48.17	15 030	51.83	<0.001
Chronic lung disease	5954	17.95	2874	48.27	3080	51.73	0.261
Diabetes mellitus	15 585	46.99	7308	46.89	8277	53.11	<0.001
Procedure characteristics							
CAD presentation							<0.001
No symptoms, no angina	2205	6.65	1198	54.33	1007	45.67	
Stable angina	6021	18.15	3055	50.74	2966	49.26	
Unstable angina	15 874	47.86	7434	46.83	8440	53.17	
NSTEMI	5955	17.96	2847	47.81	3108	52.19	
STEMI or equivalent	2396	7.22	1324	55.26	1072	44.74	
Previous PCI	20 071	60.51	9130	45.49	10 941	54.51	<0.001
Previous CABG	12 096	36.47	5091	42.09	7005	57.91	<0.001
Diagnostic Cath status							<0.001
Elective	13 771	49.09	6723	48.82	7048	51.18	
Urgent	11 399	40.64	5276	46.28	6123	53.72	
Emergency	2871	10.23	1537	53.54	1334	46.46	
Salvage	11	0.04	9	81.82	2	18.18	
Procedure year							0.335
2009	3712	11.19	1826	49.19	1886	50.81	
2010	8046	24.25	3895	48.41	4151	51.59	
2011	7010	21.13	3378	48.19	3632	51.81	
2012	7243	21.83	3578	49.40	3665	50.60	
2013	7163	21.59	3555	49.63	3608	50.37	
PCI status							<0.001
Elective	16 137	48.66	8003	49.59	8134	50.41	
Urgent	14 055	42.38	6629	47.16	7426	52.84	
Emergency	2944	8.88	1578	53.60	1366	46.40	
Salvage	25	0.08	18	72.00	7	28.00	
Multivessel disease	21 553	64.97	11 002	51.05	10 551	48.95	<0.001
Repeat PCI							
Time from index to repeat PCI							<0.001
≤30 d	2248	6.78	1021	45.42	1227	54.58	
31 to 90 d	4943	14.90	3090	62.51	1853	37.49	
>90 d	25 983	78.32	12 121	46.65	13 862	53.35	
Vessel characteristics							
No. of stents placed, median (IQR)	1.00	(1.00–2.00)	1.00		1.00		<0.001
Sum of stent diameter, median (IQR)	3.00	(2.50–3.50)	3.00		3.00		0.472
Sum of stent length, median (IQR)	23.00	(15.00–36.00)	23.00		23.00		<0.001
Stent length categorized							<0.001
Short (≤16 mm)	10 128	30.56	5034	49.70	5094	50.30	
Medium (>16–28 mm)	11 218	33.85	5690	50.72	5528	49.28	
Long (>28 mm)	11 790	35.58	5488	46.55	6302	53.45	
Mean vessel stenosis before Tx, median (IQR)	90.00	(80.00–95.00)	90.00		90.00		0.009
Previously treated lesion	6720	20.26	2414	35.92	4306	64.08	<0.001
Among previously treated lesions							
Previously treated lesion time frame							<0.001
<1 mo	258	3.85	105	40.70	153	59.30	
1 to 5 mo	1073	16.00	338	31.50	735	68.50	
6 to 12 mo	1077	16.06	338	31.38	739	68.62	
1 to 2 y	1046	15.60	363	34.70	683	65.30	
>2 y	2679	39.94	1038	38.75	1641	61.25	
Time unknown	574	8.56	224	39.02	350	60.98	
Treated with stent	6295	93.76	2229	35.41	4066	64.59	0.001
In‐stent restenosis	5875	93.42	2062	35.10	3813	64.90	0.040
In‐stent thrombosis	538	8.56	220	40.89	318	59.11	0.005
Lesion in graft							<0.001
Not in graft	27 675	83.46	14 066	50.83	13 609	49.17	
Vein	5106	15.40	1998	39.13	3108	60.87	
LIMA graft	253	0.76	105	41.50	148	58.50	
Other artery	124	0.37	58	46.77	66	53.23	
Lesion complexity							<0.001
Non‐high/non‐C	13 558	40.89	6805	50.19	6753	49.81	
High/C	19 600	59.11	9418	48.05	10 182	51.95	
Maximum lesion length, median (IQR)	18.00	(12.00–26.00)	18.00		18.00		<0.001
Thrombus present	3004	9.06	1546	51.46	1458	48.54	0.004
Bifurcation lesion	4082	12.31	1905	46.67	2177	53.33	0.002
Hospital characteristics							
Hospital location							0.006
Rural	4293	12.94	2165	50.43	2128	49.57	
Suburban	10 140	30.57	5036	49.66	5104	50.34	
Urban	18 741	56.49	9031	48.19	9710	51.81	
Profit type							0.828
Government	409	1.23	195	47.68	214	52.32	
Private/community	29 092	87.70	14 249	48.98	14 843	51.02	
University	3673	11.07	1788	48.68	1885	51.32	
Teaching Hospital	15 537	46.83	7448	47.94	8089	52.06	<0.001
PCI count, median (IQR)	850.00	(500–1347)	835.00		864.50		<0.001

CABG indicates coronary artery bypass graft; CAD, coronary artery disease; Cath, catheterization; HF, heart failure; IQR, interquartile range; LIMA, left internal mammary artery; MI, myocardial infarction; NSTEMI, non–ST‐segment–elevation myocardial infarction; NYHA, New York Heart Association; PCI, percutaneous coronary intervention; STEMI, ST‐segment–elevation myocardial infarction; Tx, treatment.

**Table 3:** Patient, Procedural, Vessel, and Hospital Characteristics of PCIs With Attributed AMI Events

	Overall		Not Attributed		Attributed		*P* Value
	No.	%	No.	Row %	No.	Row %	
N procedures	16 176		11 730	72.51	4446	27.49	
Demographics							
Age, y, mean (SD)	74.91	6.79	75.10		74.39		<0.001
Sex: female	6549	40.49	4770	72.84	1779	27.16	0.451
Race							0.364
White	14 386	88.93	10 427	72.48	3959	27.52	
Black	1219	7.54	872	71.53	347	28.47	
Asian	280	1.73	210	75.00	70	25.00	
Other	291	1.80	221	75.95	70	24.05	
Cardiovascular history							
Previous MI	7199	44.52	5059	70.27	2140	29.73	<0.001
Previous HF	4108	25.41	3039	73.98	1069	26.02	0.015
Cerebrovascular disease	3784	23.41	2712	71.67	1072	28.33	0.183
Peripheral arterial disease	4012	24.82	2867	71.46	1145	28.54	0.086
NYHA class (among those with HF in past 2 wk)							0.640
I	248	8.50	180	72.58	68	27.42	
II	766	26.24	587	76.63	179	23.37	
III	1170	40.08	882	75.38	288	24.62	
IV	735	25.18	554	75.37	181	24.63	
Cardiomyopathy or left ventricular systolic dysfunction	2671	16.52	1955	73.19	716	26.81	0.389
Other clinical history							
Current/recent smoker (w/in 1 y)	2312	14.30	1688	73.01	624	26.99	0.563
Hypertension	14 749	91.21	10 641	72.15	4108	27.85	<0.001
Dyslipidemia	13 746	85.06	9860	71.73	3886	28.27	<0.001
Chronic lung disease	3556	22.00	2623	73.76	933	26.24	0.002
Diabetes mellitus	8446	52.22	6012	71.18	2434	28.82	0.060
Procedure characteristics							
CAD presentation							<0.001
No symptoms, no angina	944	5.84	696	73.73	248	26.27	
Stable angina	1860	11.50	1345	72.31	515	27.69	
Unstable angina	6068	37.52	4194	69.12	1874	30.88	
NSTEMI	5126	31.70	3794	74.01	1332	25.99	
STEMI or equivalent	1911	11.82	1511	79.07	400	20.93	
Previous PCI	9153	56.60	6339	69.26	2814	30.74	<0.001
Previous CABG	6035	37.32	4137	68.55	1898	31.45	<0.001
Diagnostic Cath status							<0.001
Elective	4563	33.33	3146	68.95	1417	31.05	
Urgent	6973	50.93	5051	72.44	1922	27.56	
Emergency	2148	15.69	1662	77.37	486	22.63	
Salvage	7	0.05	6	85.71	1	14.29	
Procedure year							0.149
2009	1543	9.54	1130	73.23	413	26.77	
2010	3562	22.02	2622	73.61	940	26.39	
2011	3449	21.32	2472	71.67	977	28.33	
2012	3739	23.11	2733	73.09	1006	26.91	
2013	3883	24.00	2773	71.41	1110	28.59	
PCI status							<0.001
Elective	5484	33.92	3855	70.30	1629	29.70	
Urgent	8434	52.16	6126	72.63	2308	27.37	
Emergency	2231	13.80	1728	77.45	503	22.55	
Salvage	20	0.12	16	80.00	4	20.00	
Multivessel disease	10 733	66.35	7729	72.01	3004	27.99	0.044
Repeat PCI							
Time from index to repeat PCI							<0.001
≤30 d	3537	21.87	2741	77.50	796	22.50	
31 to 90 d	3013	18.63	2410	79.99	603	20.01	
>90 d	9626	59.51	6579	68.35	3047	31.65	
Vessel characteristics							
No. of stents placed, median (IQR)	1.00	(1.00–2.00)	1.00		1.00		<0.001
Sum of stent diameter, median (IQR)	3.00	(2.75–5.50)	3.00		3.50		<0.001
Sum of stent length, median (IQR)	23.00	(15.00–36.00)	23.00		24.00		<0.001
Stent length categorized							<0.001
Short (≤16 mm)	5038	31.17	3747	74.37	1291	25.63	
Medium (>16–28 mm)	5486	33.94	4026	73.39	1460	26.61	
Long (>28 mm)	5640	34.89	3946	69.96	1694	30.04	
Mean vessel stenosis before Tx, median (IQR)	90.00	(80.00–95.00)	90.00		90.00		0.104
Previously treated lesion	3027	18.72	1923	63.53	1104	36.47	<0.001
Among previously treated lesions							
Previously treated lesion time frame							0.009
<1 mo	142	4.70	99	69.72	43	30.28	
1 to 5 mo	529	17.50	314	59.36	215	40.64	
6 to 12 mo	457	15.12	286	62.58	171	37.42	
1 to 2 y	408	13.50	259	63.48	149	36.52	
>2 y	1209	39.99	802	66.34	407	33.66	
Time unknown	278	9.20	159	57.19	119	42.81	
Treated with stent	2828	93.49	1793	63.40	1035	36.60	0.657
In‐stent restenosis	2607	92.25	1649	63.25	958	36.75	0.546
In‐stent thrombosis	326	11.55	213	65.34	113	34.66	0.434
Lesion in graft							<0.001
Not in graft	13 257	81.98	9807	73.98	3450	26.02	
Vein	2725	16.85	1796	65.91	929	34.09	
LIMA graft	119	0.74	72	60.50	47	39.50	
Other artery	70	0.43	51	72.86	19	27.14	
Lesion complexity							<0.001
Non‐high/non‐C	6397	39.58	4741	74.11	1656	25.89	
High/C	9764	60.42	6977	71.46	2787	28.54	
Maximum lesion length, median (IQR)	18.00	(12.00–26.00)	18.00		18.00		<0.001
Thrombus present	2011	12.45	1523	75.73	488	24.27	<0.001
Bifurcation lesion	2022	12.51	1428	70.62	594	29.38	0.041
Hospital characteristics							
Hospital location							0.024
Rural	2402	14.85	1796	74.77	606	25.23	
Suburban	4798	29.66	3473	72.38	1325	27.62	
Urban	8976	55.49	6461	71.98	2515	28.02	
Profit type							0.394
Government	165	1.02	117	70.91	48	29.09	
Private/community	14 031	86.74	10 153	72.36	3878	27.64	
University	1980	12.24	1460	73.74	520	26.26	
Teaching Hospital	8090	50.01	5898	72.90	2192	27.10	0.266
PCI count, median (IQR)	800.00	(476–1300)	795.00		803.00		0.011

AMI indicates acute myocardial infarction; CABG, coronary artery bypass graft; CAD, coronary artery disease; Cath, catheterization; HF, heart failure; IQR, interquartile range; LIMA, left internal mammary artery; MI, myocardial infarction; NSTEMI, non–ST‐segment–elevation myocardial infarction; NYHA, New York Heart Association; PCI, percutaneous coronary intervention; STEMI, ST‐segment–elevation myocardial infarction; Tx, treatment.

## Discussion

Only half of all PCIs performed in the year after patients received a DES placement could be reasonably attributable to the same coronary artery as the index procedure, and even fewer were attributed to the same lesion as a previously placed stent. MIs and deaths can be reasonably attributable to a previously placed stent even less frequently. Whereas exploratory analyses showed that some patient and procedural characteristics were associated with higher rates of attribution, including the timing of when the event was observed, these findings suggest that using claims data alone for surveillance may be insufficient to ascertain stent safety. Rather, postmarket surveillance efforts would likely be strengthened by the use of complementary data sources, in addition to claims, when evaluating medical device safety.

Although real‐world data sources, such as claims and registries, are increasingly important for postmarket surveillance of medical devices,^4^ our study suggests that claims data alone may be inadequate for stent surveillance unless paired with additional data sources. The US Food and Drug Administration has recently stated its goal to be first among the world's regulatory agencies to identify and act upon safety signals, which involves supporting implementation of the National Evaluation System for health Technology to leverage real‐world data for surveillance.^19^ Claims are ubiquitous and longitudinal, but lack the granularity of registries. Registries are a key data source for surveillance. The Medical Device Epidemiology Network is focused on creation of coordinated registry networks for several device types, including cardiovascular devices, to provide evidence across a device's total product life cycle.^20^ However, registries often do not include longitudinal follow‐up data (such as the CathPCI Registry),^21^ and thus claims data are often used. Claims data have been shown to be concordant between physician‐adjudication and administrative claims for some events such as mortality and heart failure hospitalization.^22^ These real‐world data sources differ from clinical trials for stents, where specific stent‐related outcomes (such as stent thrombosis or in‐stent restenosis) or outcomes specific to the vessel that had received PCI (such as TVR) are ascertained and independently adjudicated to determine whether they meet criteria for standardized definitions.^18^ However, costs, complexity, and duration make performing clinical trials infeasible to generate evidence in some circumstances^23^; therefore, real‐world data sources will continue to be increasingly leveraged to provide evidence of benefits and risks of therapies,^21^ and we need to understand how to use claims and registries to refine estimates of device safety. Furthermore, some rare adverse events may never be detected in clinical trials, given that trials include fewer patients than when devices are used in real‐world clinical practice as well as shorter follow‐up durations for devices—which may be implanted lifelong.

Although claims are the most ubiquitous source of longitudinal real‐world data and offer opportunity with their size and completeness of the study populations, they also have important limitations when used for stent surveillance, or any other medical device surveillance. First, they may not always be accurate.^24^ One study showed that claims diagnoses codes for acute MI had a 94% positive predictive value for the same diagnosis when compared with electronic health record data.^25^ When compared with a clinical trial with end‐point adjudication, the kappa statistic was 0.76 for acute MI identified in *International Classification of Diseases*,* Ninth Revision* (*ICD‐9*) claims.^26^ Second, they lack granular detail about PCI location. *International Classification of Diseases*,* Tenth Revision* (*ICD‐10*) codes came into use in the United States from October 2015 and include 5 times as many diagnoses and 18 times as many procedures as the previously used *ICD‐9* codes, including greater detail about the number of sites in which stents are placed, stent restenosis, stent thrombosis, and stent fracture. However, *ICD‐10* codes still do not provide information about which coronary artery receives PCI.^27^ The possible exception is STEMI, for which *ICD‐10* codes detail the level of the culprit coronary artery which, presumably, would receive intervention. However, 50% of patients with STEMI have multivessel disease,^28^ and other coronary arteries may also receive stent placement during primary PCI for STEMI based on guideline recommendations.^29^ This means that although the utility of claims data identified in our study may improve with the transition to *ICD‐10*, the extent of improvement requires further study. Third, claims data are currently available in finalized form only after a ≈2‐year delay; to be used more effectively, they will also need to be made available more quickly. Fourth, and most critically for identifying stents of a specific manufacturer or model, claims data do not include unique device identifiers for medical devices. Unique device identifiers are barcodes that contain information about a device manufacturer, model, description, and other characteristics.^30^, ^31^ If unique device identifiers become included into claims data, specific stents and other implanted medical devices could be tracked longitudinally for surveillance purposes.^32^

Although linking claims to the CathPCI Registry helps understand whether repeat PCI is TVR, this approach is still insufficient to comprehensively understand the multiple clinical factors that determine stent safety. Patient, physician, and hospital characteristics are associated with usage of certain stents and adverse events; this means that risk standardization is necessary for surveillance. However, the lack of detailed clinical data when using administrative claims as the longitudinal data source makes this inadequate at the patient level. For example, patient adherence to thienopyridines declines within 1 year, thus increasing the risk of stent thrombosis.^33^ Therefore, surveillance of DES using real‐world data could be made more robust through combination with additional data sources such as electronic health records, pharmacy claims data, and patient‐reported data.

Whereas mortality is arguably the most important clinical end point for DES safety, not unexpectedly, we could only determine that ≈1% of deaths identified in claims could be reasonably attributed to a complication within the same coronary artery in which the index PCI was performed. Because CathPCI Registry data are available for patients with documented PCI and sometimes diagnostic coronary angiography, we cannot ascertain the reason for death identified in claims for the vast majority of patients because these patients died outside of the hospital or even when hospitalized and did not receive coronary angiography and/or PCI. Some of these deaths could have been stent‐related, such as stent thrombosis leading to acute MI and sudden cardiac death. When patients had a documented repeat PCI but died, 71.4% were in the same coronary artery as was initially stented. As with death, there is incomplete attribution of MIs to previously placed stents, such as patients who experienced an MI and did not receive medical care, did not receive diagnostic coronary angiography, or did not receive PCI. Real‐world safety evaluations will often use death and MI as end points, given their clinical significance and the ease with which they can be ascertained from nationally representative claims data, but will also lack the capacity for adjudication as is done in clinical trials. Therefore, our finding that only a small proportion represent adverse events that can be attributable to previously placed stents means that these claims‐based end points can only serve as a signal that must be further evaluated and refined with complementary data sources.

Our findings may be considered in the context of several limitations. First, we excluded patients receiving multivessel PCI, which is performed in a substantial minority of cases. When multiple coronary arteries receive PCI, attribution of stent‐related safety events will be more difficult. Second, we excluded patients with repeat procedures within 60 days unless they had an MI code or complication code. Although we presumed that most of these patients were receiving staged PCI,^17^ we still may have missed some stent‐related complications, particularly if a repeat PCI occurred within the same coronary artery as the index PCI. Third, we did not include in‐hospital stent‐related adverse events, which are nearly always stent thrombosis. Claims data preclude distinguishing index from repeat PCI within a given hospitalization. Fourth, we did not examine coronary artery bypass grafting in longitudinal follow‐up, which may infrequently be a reason for revascularization after a stent‐related adverse event. Fifth, we did not examine patients longitudinally in the NCDR CathPCI Registry and then attempt to locate a corresponding CMS claim. Sixth, although the CathPCI Registry captures granular information on the coronary segment in which a device is used, these data are inconsistently reported and unlikely to be reliable. For that reason, we instead focused on the less‐granular TVR, making our estimates a “better” case scenario for attribution of adverse events to a previously placed stent. Seventh, we did not evaluate adverse events occurring because of operator‐level variation in performance of PCI. Finally, our findings are applicable only to coronary stents, given that a comprehensive national registry exists to capture PCI and the expected adverse events can be captured within claims data.

In conclusion, by linking longitudinal claims data to a comprehensive national registry of PCIs multiple times, we found that approximately half of repeat PCIs within 1 year occur in the same coronary artery as the initial PCI with DES placement, indicating a DES‐related adverse event. MI and death, although more clinically important, could be attributed much less often to the same coronary artery as the index PCI. These findings suggest that using claims data for surveillance of DESs, even when linked to a national PCI registry, may be insufficient. As momentum grows to leverage real‐world data for medical device surveillance, these limitations will need to be surmounted through novel strategies to bring together complementary data sources to inform a robust postmarket surveillance system.

## Sources of Funding

This project was jointly funded by the US Food and Drug Administration (FDA) and Medtronic, Inc. to develop methods for postmarket surveillance of medical devices (U01FD004585). Members of the sponsoring organizations contributed directly to the project, participating in study conception and design, analysis and interpretation of data, and critical revision of the manuscript; the authors made the final decision to submit the manuscript for publication. In addition, the project was approved by, but did not receive financial support from, the American College of Cardiology's NCDR. The NCDR Research and Publications Committee reviewed the final manuscript before submission, but otherwise had no role in the design, conduct, or reporting of the study. Drs Masoudi and Shaw receive support from the American College of Cardiology for roles within the NCDR. Dr Curtis receives support from the American College of Cardiology and has equity interest in Medtronic. Dr Dhruva is supported by the Department of Veterans Affairs. The authors assume full responsibility for the accuracy and completeness of the ideas presented, which do not represent the views of the Department of Veterans Affairs or any other supporting institutions.

## Disclosures

In the past 36 months, Dr Ross has received research support through Yale University from the US Food and Drug Administration to establish the Yale–Mayo Clinic Center for Excellence in Regulatory Science and Innovation (CERSI) program (U01FD005938), from the Laura and John Arnold Foundation to support the Collaboration for Research Integrity and Transparency (CRIT) at Yale, from the Agency for Healthcare Research and Quality (R01HS022882), and from the Blue Cross Blue Shield Association to better understand medical technology evidence generation; Drs Krumholz, Ross, and Ms Gamble have received research support from Johnson & Johnson to develop methods of clinical trial data sharing; and Drs Curtis, Desai, Normand, Krumholz, and Ross and Mr Parzynski work under contract to the Centers for Medicare and Medicaid Services (CMS) to develop and maintain performance measures that are used for public reporting. Dr Yeh receives research support from the National Heart, Lung, and Blood Institute (R01HL136708) and reports grant support from Abiomed, AstraZeneca, and Boston Scientific and consulting fees from Abbott, Boston Scientific, Medtronic, and Teleflex. Dr Kuntz is an employee of Medtronic, Inc. Dr Marinac‐Dabic is an employee of the US Food and Drug Administration. Dr Krumholz chairs a cardiac scientific advisory board for UnitedHealth; is a participant/participant representative of the IBM Watson Health Life Sciences Board; is a member of the Advisory Board for Element Science and the Physician Advisory Board for Aetna; and is the founder of Hugo, a personal health information platform.

## Supporting information


**Table S1.** *ICD‐9 CM*/CPT Codes Used to Identify Longitudinal Outcomes in Inpatient and Outpatient Institutional Claims for Medicare Fee‐for‐Service Beneficiaries
**Table S2.** Characteristics of Procedures With Adverse Events Within 1 Year Identified in Medicare Claims Data Linked and Not Linked to National Cardiovascular Data Registry
**Figure S1.** Attribution of adverse events identified in Medicare claims data after index drug‐eluting stent placement in patients aged ≥65 years, 2009–2013 who did not have a history of previous percutaneous coronary intervention.
