# Supplementary material for: Attribution of Adverse Events Following Coronary Stent Placement Identified Using Administrative Claims Data
Source: J Am Heart Assoc. 2020 Feb 16;9(4):e013606. doi: 10.1161/JAHA.119.013606 (PMC7070203; doi:10.1161/JAHA.119.013606)
Supplement: Supplementary file 1 — Table S1. ICD‐9 CM/CPT Codes Used to Identify Longitudinal Outcomes in Inpatient and Outpatient Institutional Claims for Medicare Fee‐for‐Service Beneficiaries Table S2. Characteristics of Procedures With Adverse Events Within 1 Year Identified in Medicare Claims Data Linked and Not Linked to National Cardiovascular Data Registry Figure S1. Attribution of adverse events identified in Medicare claims data after index drug‐eluting stent placement in patients aged ≥65 years, 2009–2013 who did not have a history of previous percutaneous coronary intervention. [file JAH3-9-e013606-s001.pdf]

SUPPLEMENTAL MATERIAL

Table S1. ICD-9 CM/CPT codes used to identify longitudinal outcomes in inpatient and outpatient institutional claims for Medicare fee-for-service beneficiaries.

Outcome	Source	Code	Definition
Repeat percutaneous coronary intervention (PCI)	ICD-9 SG	00.66	Percutaneous transluminal coronary angioplasty [PTCA]
	ICD-9 SG	36.06	Insertion of non-drug-eluting coronary artery stent(s)
	ICD-9 SG	36.07	Insertion of drug-eluting coronary artery stent(s)
	ICD-9 SG	36.09	Other removal of coronary artery obstruction
	CPT	92920	PTCA
	CPT	92921	PTCA; each additional branch of a major coronary artery
	CPT	92924	Percutaneous transluminal coronary atherectomy with angioplasty
	CPT	92925	Percutaneous transluminal coronary atherectomy, with coronary angioplasty when performed; each additional branch of a major coronary artery
	CPT	92928	Percutaneous transcatheter insertion of stent with coronary angioplasty
	CPT	92929	Percutaneous transcatheter insertion of stent with coronary angioplasty
	CPT	92933	Percutaneous transluminal coronary atherectomy, with intracoronary stent, with coronary angioplasty when performed; single major coronary artery or branch
	CPT	92934	Percutaneous transluminal coronary atherectomy with angioplasty and insertion of stent

CPT	92937	Percutaneous transluminal revascularization of coronary artery with atherectomy and insertion of stent
CPT	92938	Percutaneous transluminal revascularization of coronary artery bypass graft with angioplasty and insertion of stent
CPT	92941	Percutaneous transluminal revascularization of coronary artery bypass graft with atherectomy, angioplasty and insertion of stent
CPT	92943	Percutaneous transluminal revascularization of coronary artery bypass graft with atherectomy, angioplasty and insertion of stent
CPT	92944	Percutaneous transluminal revascularization of coronary artery bypass graft with atherectomy, angioplasty and insertion of stent
CPT	92980	Transcatheter placement of an intracoronary stent(s), percutaneous, with or without other therapeutic intervention, any method; single vessel
CPT	92981	Transcatheter placement of an intracoronary stent(s), percutaneous, with or without other therapeutic intervention, any method; each additional vessel
CPT	92982	Percutaneous transluminal coronary balloon angioplasty, single vessel
CPT	92984	Percutaneous transluminal coronary balloon angioplasty, each additional vessel
CPT	92995	Percutaneous transluminal coronary atherectomy, by mechanical or other method, with or without balloon angioplasty; single vessel

CPT	92996	Percutaneous transluminal coronary atherectomy, by mechanical or other method, with or without balloon angioplasty; each additional vessel
CPT	C9600	Percutaneous transcatheter placement of drug-eluting intracoronary stent(s), with coronary angioplasty when performed; single major coronary artery or branch
CPT	C9601	Percutaneous transcatheter placement of drug-eluting intracoronary stent(s), with coronary angioplasty when performed; each additional branch of a major coronary artery (list separately in addition to code for primary procedure)
CPT	C9602	Percutaneous transluminal coronary atherectomy, with drug-eluting intracoronary stent, with coronary angioplasty when performed; single major coronary artery or branch
CPT	C9603	Percutaneous transluminal coronary atherectomy, with drug-eluting intracoronary stent, with coronary angioplasty when performed; each additional branch of a major coronary artery (list separately in addition to code for primary procedure)
CPT	C9604	Percutaneous transluminal revascularization of or through coronary artery bypass graft (internal mammary, free arterial, venous), any combination of drug-eluting intracoronary stent, atherectomy and angioplasty, including distal protection when performed; single vessel
CPT	C9605	Percutaneous transluminal revascularization of or through coronary artery bypass graft (internal mammary, free arterial,

		venous), any combination of drug-eluting intracoronary stent, atherectomy and angioplasty, including distal protection when performed; each additional branch subtended by the bypass graft (list separately in addition to code for primary procedure)
CPT	C9606	Percutaneous transluminal revascularization of acute total/subtotal occlusion during acute myocardial infarction, coronary artery or coronary artery bypass graft, any combination of drug-eluting intracoronary stent, atherectomy and angioplasty, including aspiration thrombectomy when performed, single vessel
CPT	C9607	Percutaneous transluminal revascularization of chronic total occlusion, coronary artery, coronary artery branch, or coronary artery bypass graft, any combination of drug-eluting intracoronary stent, atherectomy and angioplasty; single vessel
CPT	C9608	Percutaneous transluminal revascularization of chronic total occlusion, coronary artery, coronary artery branch, or coronary artery bypass graft, any combination of drug-eluting intracoronary stent, atherectomy and angioplasty; each additional coronary artery, coronary artery branch, or bypass graft (list separately in addition to code for primary procedure)
CPT	G0290	Transcatheter placement of a drug-eluting intracoronary stent(s), percutaneous, with or without other therapeutic intervention, any method; single vessel

	CPT	G0291	Transcatheter placement of a drug-eluting intracoronary stent(s), percutaneous, with or without other therapeutic intervention, any method; each additional vessel
Acute myocardial infarctions (AMIs)	ICD-9 DX	410.01	AMI of anterolateral wall, initial episode of care
	ICD-9 DX	410.11	AMI of other anterior wall, initial episode of care
	ICD-9 DX	410.21	AMI of inferolateral wall, initial episode of care
	ICD-9 DX	410.31	AMI of inferoposterior wall, initial episode of care
	ICD-9 DX	410.41	AMI of other inferior wall, initial episode of care
	ICD-9 DX	410.51	AMI of other lateral wall, initial episode of care
	ICD-9 DX	410.61	True posterior wall infarction, initial episode of care
	ICD-9 DX	410.71	Subendocardial infarction, initial episode of care
	ICD-9 DX	410.81	AMI of other specified sites, initial episode of care
	ICD-9 DX	410.91	AMI of unspecified site, initial episode of care

Table S2. Characteristics of procedures with adverse events within one year identified in Medicare claims data linked and not linked to National Cardiovascular Data Registry.

	Procedures with AE		Procedures with AE not linked to NCDR with coronary artery info from PCI		Procedures with AE linked to NCDR with coronary artery info from PCI	
	#	%	#	%	#	%
**N Procedures**	61,409	100.00	32,802	53.42	28,607	46.58
**Demographics**						
Age - Mean (SD)	75.14	6.91	76.24	7.17	73.88	6.36
Sex: Female	23,408	38.12	12,909	39.35	10,499	36.70
Race						
White	55,660	90.64	29,554	90.10	26,106	91.26
Black	3,849	6.27	2,203	6.72	1,646	5.75
Asian	906	1.48	486	1.48	420	1.47
Other	994	1.62	559	1.70	435	1.52
Hispanic or Latino Ethnicity	2,413	3.94	1,376	4.20	1,037	3.63
**Cardiovascular History**						
Prior MI	24,096	39.26	13,027	39.73	11,069	38.71
Prior Heart Failure	15,438	25.15	10,435	31.83	5,003	17.50
Prior Valve Surgery/Procedure	1,719	2.80	1,038	3.17	681	2.38
Cerebrovascular Disease	13,758	22.42	7,997	24.39	5,761	20.15
Peripheral Arterial Disease	14,417	23.49	8,396	25.61	6,021	21.05

NYHA Class (among those with HF in prior 2 weeks)						
Class I	850	7.54	502	6.21	348	10.87
Class II	2,900	25.71	1,952	24.16	948	29.62
Class III	4,522	40.09	3,295	40.79	1,227	38.33
Class IV	3,007	26.66	2,329	28.83	678	21.18
Cardiomyopathy or Left Ventricular Systolic Dysfunction	10,475	17.06	6,990	21.31	3,485	12.18
Cardiogenic Shock w/in 24 Hrs	941	1.53	722	2.20	219	0.77
Cardiac Arrest w/in 24 Hrs	724	1.18	533	1.63	191	0.67
**Other Clinical History**						
Current/Recent Smoker (w/in 1 year)	8,122	13.23	4,879	14.88	3,243	11.34
Hypertension	55,484	90.39	29,411	89.71	26,073	91.18
Dyslipidemia	51,773	84.39	26,658	81.35	25,115	87.87
Currently on Dialysis	3,936	6.42	2,750	8.39	1,186	4.15
Chronic Lung Disease	14,920	24.31	9,813	29.93	5,107	17.86
Diabetes Mellitus	29,303	47.73	15,817	48.24	13,486	47.15
**Procedure Characteristics**						
CAD Presentation						
No symptom, no angina	4,620	7.52	2,727	8.31	1,893	6.62
Symptom unlikely to be ischemic	1,564	2.55	959	2.92	605	2.12
Stable angina	9,363	15.25	4,185	12.76	5,178	18.11
Unstable angina	25,962	42.29	12,225	37.27	13,737	48.03

Non-STEMI	14,044	22.87	8,905	27.15	5,139	17.97
ST-Elevation MI (STEMI) or equivalent	5,844	9.52	3,797	11.58	2,047	7.16
Prior PCI	33,562	54.67	16,163	49.29	17,399	60.83
Prior CABG	20,638	33.62	10,074	30.72	10,564	36.93
Diagnostic Cath Status						
Elective	21,599	42.08	9,693	35.87	11,906	48.98
Urgent	22,921	44.65	12,989	48.06	9,932	40.86
Emergency	6,753	13.16	4,293	15.89	2,460	10.12
Salvage	58	0.11	49	0.18	9	0.04
Procedure Year						
2009	6,439	10.49	3,181	9.70	3,258	11.39
2010	14,567	23.72	7,618	23.22	6,949	24.29
2011	12,902	21.01	6,895	21.02	6,007	21.00
2012	13,662	22.25	7,413	22.60	6,249	21.84
2013	13,839	22.54	7,695	23.46	6,144	21.48
PCI Status						
Elective	26,001	42.36	12,118	36.96	13,883	48.55
Urgent	28,315	46.13	16,140	49.23	12,175	42.58
Emergency	6,970	11.36	4,452	13.58	2,518	8.81
Salvage	92	0.15	73	0.22	19	0.07
IABP	1,136	1.85	864	2.63	272	0.95
Other Mechanical Ventricular Support	335	0.55	245	0.75	90	0.31

Multi Vessel Disease	38,654	62.95	19,959	60.85	18,695	65.35
**Vessel Characteristics**						
# of Stents Placed - Median (IQR)	1.00	(1.00-2.00)	1.00	(1.00-2.00)	1.00	(1.00-2.00)
Sum of Stent Diameter - Median (IQR)	3.00	(2.75-5.00)	3.00	(2.75-5.00)	3.00	(2.75-5.00)
Sum of Stent Length - Median (IQR)	22.00	(15.00-33.00)	22.00	(15.00-33.00)	22.00	(15.00-32.00)
Stent Length Categorized						
Small (16mm or less)	141,135	34.02	141,135	34.02	141,135	34.02
Medium (>16mm - 28mm)	146,361	35.28	146,361	35.28	146,361	35.28
Large (> 28mm)	127,332	30.70	127,332	30.70	127,332	30.70
Mean Vessel Stenosis Prior to Tx - Median (IQR)	90.00	(80.00-95.00)	90.00	(80.00-95.00)	90.00	(80.00-95.00)
Pre-Procedure TIMI Flow						
TIMI - 0	37,967	9.17	37,967	9.17	37,967	9.17
TIMI - 1	32,786	7.92	32,786	7.92	32,786	7.92
TIMI - 2	82,852	20.00	82,852	20.00	82,852	20.00
TIMI - 3	260,598	62.92	260,598	62.92	260,598	62.92
Previously Treated Lesion	50,666	12.21	50,666	12.21	50,666	12.21
Among previously Treated Lesions:						
Previously Treated Lesion Timeframe						
< 1 month	1,994	3.94	1,994	3.94	1,994	3.94
1-5 months	5,668	11.21	5,668	11.21	5,668	11.21

6-12 months	6,370	12.60	6,370	12.60	6,370	12.60
1-2 years	7,209	14.26	7,209	14.26	7,209	14.26
>2 years	25,365	50.16	25,365	50.16	25,365	50.16
Time unknown	3,959	7.83	3,959	7.83	3,959	7.83
Treated with Stent	47,152	93.19	47,152	93.19	47,152	93.19
In-stent Restenosis	43,445	92.19	43,445	92.19	43,445	92.19
In-stent Thrombosis	4,613	9.80	4,613	9.80	4,613	9.80
Lesion in Graft						
Not in graft	377,850	91.02	377,850	91.02	377,850	91.02
Vein	34,644	8.35	34,644	8.35	34,644	8.35
LIMA graft	1,854	0.45	1,854	0.45	1,854	0.45
Other artery	795	0.19	795	0.19	795	0.19
Lesion Complexity						
Non-High/Non-C	190,068	45.80	190,068	45.80	190,068	45.80
High/C	224,936	54.20	224,936	54.20	224,936	54.20
Maxmium Lesion Length - Median (IQR), mm	18.00	(12.00-24.00)	18.00	(12.00-24.00)	18.00	(12.00-24.00)
Thrombus Present	41,432	9.98	41,432	9.98	41,432	9.98
Bifurcation Lesion	50,592	12.19	50,592	12.19	50,592	12.19

NCDR: National Cardiovascular Data Registry

PCI: Percutaneous Coronary Intervention

CABG: Coronary Artery Bypass Graft

CAD: Coronary Artery Disease

HF: Heart Failure

Hrs: Hours

IABP: Intra-Aortic Balloon Pump

LIMA: Left Internal Mammary Artery

MI: Myocardial Infarction

NYHA: New York Heart Association

STEMI: ST-Elevation MI

TIMI: Thrombolysis In Myocardial Infarction

Tx: Treatment

Figure S1. Attribution of adverse events identified in Medicare claims data after index drug-eluting stent placement in patients 65 years and older, 2009-2013 who did not have a history of prior percutaneous coronary intervention.

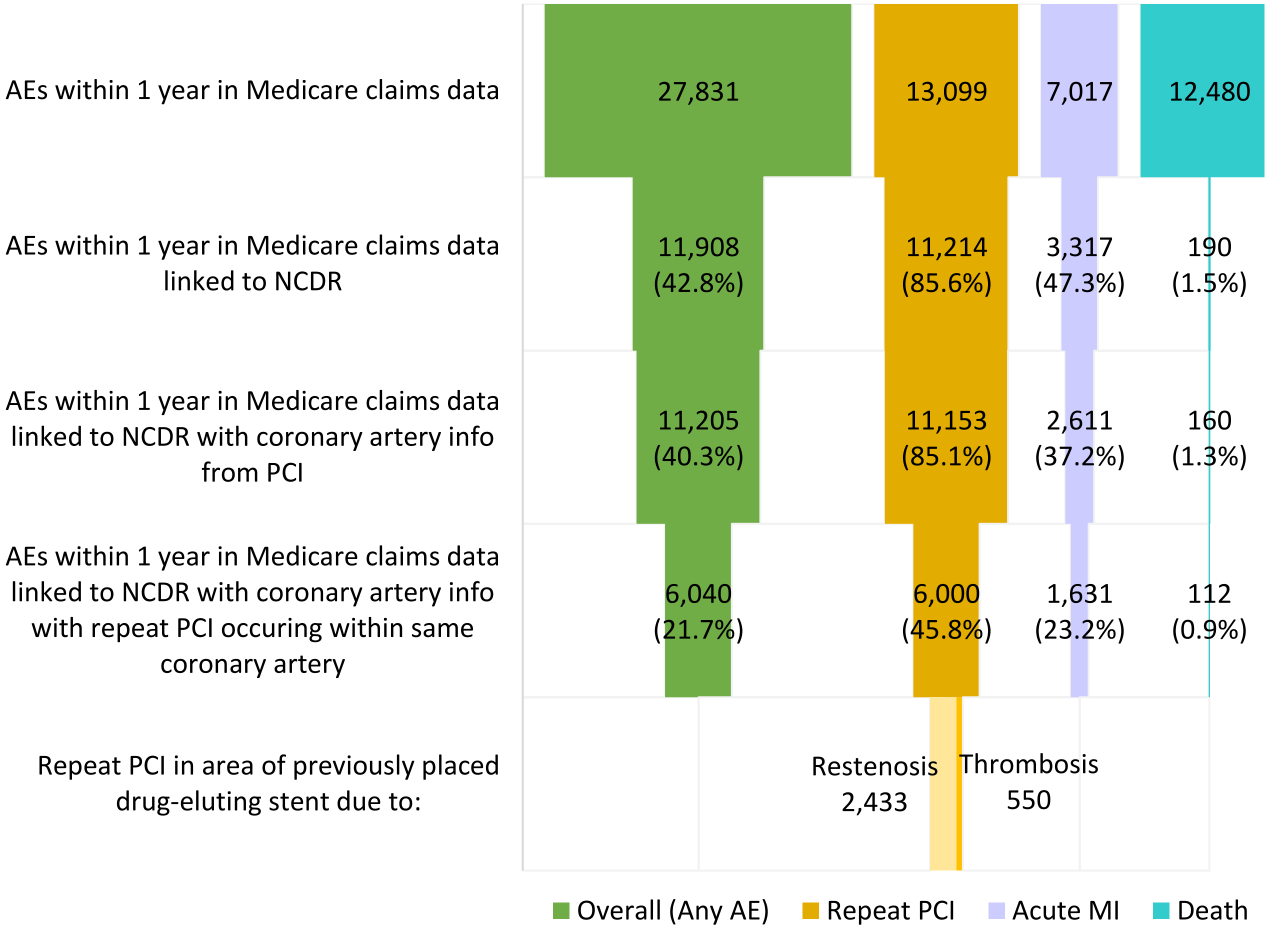

AEs, adverse events; MI, myocardial infarction; NCDR, National Cardiovascular Data Registry; PCI, percutaneous coronary intervention.
